# Intra-host evolution of cell-fusing agent virus following acute infection in *Aedes aegypti* mosquito

**DOI:** 10.1093/ve/veaf079

**Published:** 2025-09-30

**Authors:** Mohammad Mosleh Uddin, Yasutsugu Suzuki, Dan Joseph C Logronio, Kozo Watanabe

**Affiliations:** Center for Marine Environmental Studies (CMES), Ehime University, Bunkyo-cho 3, Matsuyama, Ehime, 790-8577, Japan; Graduate School of Science and Engineering, Ehime University, Bunkyo-cho 3, Matsuyama, Ehime, 790-8577, Japan; Department of Biochemistry and Molecular Biology (BMB), Faculty of Life Science, Mawlana Bhashani Science and Technology University (MBSTU), Santosh, Tangail 1902, Bangladesh; Center for Marine Environmental Studies (CMES), Ehime University, Bunkyo-cho 3, Matsuyama, Ehime, 790-8577, Japan; Center for Marine Environmental Studies (CMES), Ehime University, Bunkyo-cho 3, Matsuyama, Ehime, 790-8577, Japan; Graduate School of Science and Engineering, Ehime University, Bunkyo-cho 3, Matsuyama, Ehime, 790-8577, Japan; Center for Marine Environmental Studies (CMES), Ehime University, Bunkyo-cho 3, Matsuyama, Ehime, 790-8577, Japan

**Keywords:** insect specific virus, population size, genetic diversity, genetic structure, genetic drift

## Abstract

While intra-host evolution of arboviruses in mosquitoes has been documented, studies of insect-specific viruses (ISVs) remain limited. This study examines evolutionary patterns [i.e. evolutionary process, mutational types (synonymous/nonsynonymous)] of the cell-fusing agent virus (CFAV), an ISV that infects adult *Aedes aegypti*, over a period of 21 days post-infection (dpi), with a focus on the relationship between viral population dynamics and genetic diversity. High-throughput sequencing of amplification products covering the entire viral genome revealed a significant positive correlation of CFAV genetic diversity with viral population size and natural selection (${d}_N$/${d}_S$). Notably, diversity for both synonymous and nonsynonymous single nucleotide variant (SNV) sites displayed a positive correlation with population size and natural selection suggesting that genetic drift and purifying selection contribute to the overall outcome of genetic diversity. Additionally, we confirmed that smaller viral population sizes lead to greater temporal changes in genetic structure, particularly evident between Day 1 dpi and Day 3 dpi when genetic drift was most pronounced. We found that non-structural (NS) genes accumulated a higher frequency of synonymous SNV sites than structural genes, likely due to reduced selection pressure acting on NS genes. In contrast, structural genes, particularly the E gene, are likely to exhibit strong selective pressure, as indicated by a significant frequency of nonsynonymous SNV sites. Overall, this study elucidated the evolutionary patterns of CFAV, highlighting the roles of reduced genetic drift as influenced by population size and purifying selection in shaping the overall genetic diversity—and possibly adaptive evolution within structural genes, such as the E gene.

## Introduction

Virus populations in mosquitoes undergo evolution through mechanisms such as mutation, genetic drift, and natural selection (Moya et al. 2004, Geoghegan and Holmes 2018). While mosquitoes are recognized as vectors for various types of viruses, including arboviruses like dengue virus (DENV), the majority of virus species found in mosquitoes are classified as insect-specific viruses (ISVs), which are exclusively found in insects or other arthropods (Nasar et al. 2012, Calisher and Higgs 2018). ISVs are believed to be vertically transmitted from the carrier female mosquito to her offspring through transovarian transmission, thereby maintaining their life cycle (Cook et al. 2006, Lutomiah et al. 2007, Saiyasombat et al. 2011, Nag and Efner 2024). Although the intra-host evolution of arboviruses in mosquitoes has been documented in studies involving DENV (Lequime et al. 2016, Ko et al. 2018) and West Nile Virus (WNV) (Grubaugh et al. 2016), there is a notable lack of research specifically focused on ISVs. Understanding the evolution of ISVs may also provide insights into viral adaptability, vertical transmission, and potential host switching (Parameswaran et al. 2017, Ko et al. 2018, Ngo et al. 2019).

Understanding the evolutionary forces at play during the rapid replication of RNA viruses is a fundamental question in the study of ISVs. In a suitable host experiencing acute infection, RNA viruses undergo rapid replication cycles, which can lead to increased genetic variation due to the error-prone activity of RNA-dependent RNA polymerase (Moya et al. 2004, Geoghegan and Holmes 2018, Sanjuán and Domingo-Calap 2021). Furthermore, genetic drift can theoretically reduce genetic diversity in RNA viruses when population sizes are small (Moya et al. 2004, Dolan et al. 2018, Retel et al. 2019, Sanjuán and Domingo-Calap 2021). Immediately following acute infection, the viral population is limited in size, leading to diminished genetic diversity due to genetic drift. However, as replication progresses and the population expands, the influence of genetic drift decreases, resulting in an increase in genetic diversity driven by replication errors. It is important to note that while genetic drift reduces diversity within individual viral populations, it can simultaneously promote differentiation between populations. Consequently, after acute infection, the genetic structure of the viral population is expected to fluctuate significantly, stabilizing only after the population size increases (Geoghegan and Holmes 2018). A comprehensive understanding of the relationship between viral population dynamics within hosts and neutral evolution necessitates an examination of the balance between increased genetic diversity due to replication errors and decreased diversity due to genetic drift (Sanjuán and Domingo-Calap 2021). Nonetheless, the evolution of viruses within hosts may involve not only neutral processes, but also natural selection based on mutational types such as synonymous and nonsynonymous mutation (Cuevas et al. 2012). Synonymous mutations, which do not change the protein sequence, are often considered neutral, allowing genetic diversity without immediate functional consequences (Gojobori et al. 1994, Domingo 2020). In contrast, nonsynonymous mutations, which alter the protein sequence, are under stronger selective pressure (Gojobori et al. 1994). Therefore, the comparison of mutational types in viral genomes is essential for understanding overall evolutionary processes in viruses during population growth. However, empirically, there is limited research on the effect of population size on genetic diversity and the temporal variability of population genetic structure during the process of viral replication in the host, and none specifically on ISVs.

Furthermore, for RNA viruses to successfully enter and replicate within host cells, they must bind to host cell surface receptors and co-receptors with viral membrane (M)-associated proteins (structural proteins), while delivering their genome to the host (Cruz-Oliveira et al. 2015, Reyes et al. 2021). During this entry process, viruses face selective pressure (Öhlund et al. 2019), driving mutations that facilitate infection (Chaguza et al. 2023). Arboviruses such as DENV enter host cells through the viral envelope protein (E) (Reyes-del Valle and del Angel 2004, Acosta et al. 2008) and are therefore subject to strong selection pressures during host recognition. For example, Parameswaran et al. (2017) identified dominant DENV variants in human clinical samples that carried nonsynonymous mutations in the M protein and E protein. Their findings suggest that immune selection drives mutations in these genes, helping the virus to evade host immunity. However, infection dynamics following acute ISV infection in the host cell, and the corresponding immune response remain limited. Hypothetically, ISV infection in mosquito host cells should follow trends similar to those observed in other RNA viruses (Cruz-Oliveira et al. 2015, Reyes et al. 2021) which suggest that mutation types might vary in the structural and non-structural (NS) genes during the infection of the host cells and/or cell types. Therefore, examining the accumulation of mutation types in structural and NS genes is crucial for understanding selection pressures during viral infection dynamics.

The first insect-specific virus discovered was cell-fusing agent virus (CFAV) in an *Aedes (Ae.) aegypti* cell line in 1975 (Stollar and Thomas 1975). Like other Orthoflavivirus, the genomic structure of CFAV consists of a long polyprotein gene within a 5′-untranslated region (5′-UTR) and a 3′-UTR. The genes in polyproteins such as capsid (C), pre-M (PrM), M, and E are involved in shaping the viral structure and are therefore referred to as structural proteins. Other genes such as NS1, NS2A, NS2B, NS3, NS4A, NS4B, 2K, and NS5 are involved in viral replication and packaging and are therefore referred to as NS proteins. CFAV is a geographically dispersed ISV that is commonly found in *Ae. aegypti* mosquitoes (Baidaliuk et al. 2020). Numerous studies have been conducted with CFAV across diverse platforms, including its potential use in controlling medically important arboviruses (Baidaliuk et al. 2019), its impact on host fitness (Suzuki et al. 2024), vertical and/or horizontal transmission in the host (Logan et al. 2022, Zhou et al. 2023), antiviral function of CFAV-derived endogenous viral elements (EVEs) (Goic et al. 2016, Suzuki et al. 2020), and characterization of CFAV-derived DNA that may be a precursor of the EVEs (Uddin et al. 2024). All these multifaceted studies on CFAV allowed us to consider it as a representative model for ISV evolution studies.

In this study, we aimed to investigate the intra-host evolutionary processes of CFAV during replication kinetics in acutely infected *Ae. aegypti* mosquitoes, which serve as an experimental model organism for insect specific flaviviruses. To achieve this, we employed a whole-genome deep sequencing framework based on PCR amplicon products, which allows comprehensive coverage of the entire viral genome at an appropriate depth (Grubaugh et al. 2019, Ko et al. 2020). We tested the following three hypotheses to explore the evolutionary effect of viral population size on genetic diversity during CFAV replication kinetics: (i) intra-host genetic diversity of CFAV increases with population size, (ii) when the population size is small, the temporal change in population genetic structure is large; however, as the population size increases, this change becomes smaller, and (iii) structural genes have a higher number of nonsynonymous mutations than NS genes. This study represents the first experimental evolutionary study of an ISV in mosquitoes.

## Materials and methods

### Mosquito infection and cell-fusing agent virus RNA quantification

A laboratory strain of *Ae. aegypti*, previously engineered by Suzuki et al. (2020)  *via* CRISPR/Cas9-mediated deletion of CFAV-derived EVEs was selected in this study. This mosquito strain was originally established from an isofemale line of *Ae. aegypti* collected in Kamphaeng Phet Province, Thailand (Fansiri et al. 2013). This mosquito was used to exclude any antiviral effects and/or artefact effects in the next generation sequencing (NGS) reads caused by the EVEs in the downstream analysis (Suzuki et al. 2020, Suzuki et al. 2024). Three to 4 days after emergence, adult female mosquitoes were cold shock anesthetized, placed on a cold plate, and intrathoracically inoculated with ~ 3.5 × 10^4^ CFAV RNA copies (35 nl of CFAV stock) using a nanoinjector (Nanoject III, Drummond Scientific, Broomall, PA, USA). The injected mosquitoes were then reared in the standard environmental cabinet at 28°C and 80% relative humidity under a 12-h photoperiodic condition. Following injection, mosquitoes were collected at 0, 1, 3, 5, 7, 10, 14, and 21 days post injection (dpi).

At each time point, five to eight mosquitoes were initially screened to observe the viral growth curve and estimated population size. From these, three individuals were randomly selected as representative samples for deep sequencing. CFAV RNA copies were estimated using slightly modified protocols published in Uddin et al. (2024). Briefly, total RNA was extracted from individual mosquitoes using a Qiagen RNeasy mini kit following DNase I treatment to exclude potential DNA contamination according to the manufacturer’s protocol and eluted with 30 μl of RNase-free water. cDNA was synthesized from 15 μl of total RNA using SuperScript IV VILO Master Mix (Invitrogen). To quantify absolute CFAV RNA copies per mosquito, CFAV NS3-specific ssRNA was amplified with primers containing the T7 promoter sequence using the MEGAscript T7 Transcription Kit (Thermo Fisher Scientific, USA), followed by reverse transcription to generate a standard curve. Real-time reverse transcription quantitative polymerase chain reaction (RT-qPCR) was performed based on a serial dilution of CFAV-NS3 RNA using iTaq Universal SYBER Green Supermix (Bio-Rad, USA). Primer sequences are listed in the Supplementary Material, S1 File (XLSX). Note that equal volumes of cDNA were used in the initial single-plex PCR to generate amplicons in order to standardize the viral load.

### Cell-fusing agent virus whole genome primer design and their PCR efficiency validation

To investigate the evolutionary dynamics of CFAV during population expansion, we employed a whole-genome amplicon-based sequencing framework. We designed 35 overlapping primers using the PrimalScheme protocol (Quick et al. 2017). The primers generated amplicons ranging from 380 to 420 bp, the sequences of which are provided in the electronic Supplementary Material, S1 File (XLSX). The PCR efficiency of all primers was validated and optimized by mixing with 0.025 μl of cDNA, 0.3 μM of each primer, and 0.5 mM of dNTPs in a 15 μl PCR reaction containing Q5 high-fidelity DNA polymerase (0.02 U/μl) (M0491S, New England Biolab). The PCR conditions included an initial temperature of 98°C for 30 s, followed by 35 cycles of denaturation at 98°C for 10 s, annealing at 67°C for 30 s, and extension at 72°C for 40 s, with a final extension at 72°C for 2 min. All PCR products were visualized on an agarose gel, with images provided in the supplementary material (Supplementary Fig. 1).

We performed biological triplicate sampling of each time point, including a pre-infected CFAV sample, referred to as the ancestral sample. One representative of the 35 amplified PCR products from a CFAV-injected mosquito was Sanger sequenced to confirm correct amplification and to assess CFAV genome coverage and overlap. PCR products were treated with ExoSAP-IT to remove contaminants prior to Sanger sequencing.

### Amplicon-based sequencing library construction

To achieve complete genome coverage of CFAV by Illumina sequencing, PCR was performed for all 35 primer pairs across all mosquito samples under uniform conditions. We pooled 10 μl of each amplicon generated from the 35 primers per CFAV-infected mosquito prior to library preparation. The pooled amplicons were purified using Agencourt AMPure XP magnetic beads (Beckman Coulter, USA) with a double size selection ratio of 0.85–0.55 to remove contaminants, including primer dimers. The quality of the purified amplicons was checked using a DNA bioanalyzer (Agilent 2100 Bioanalyzer system) with a high-sensitivity DNA kit (Agilent Technologies, product number: 5067-4627, US). Libraries were prepared using the Illumina DNA Prep–(M) Tagmentation Kit according to the manufacturer’s instructions, with a DNA input of 120 ng per library. Libraries were quantified using the KAPA library quantification kit (Illumina) and quality checked using a DNA bioanalyzer (Agilent 2100 Bioanalyzer system). An equimolar pool of individual libraries was prepared for sequencing, generating paired-end 300 nt reads using the MiSeq V3 600 cycle kits (Illumina, USA) at a concentration of 10 pM with 10% PhiX. Three technical replicates were performed for a pre-infected CFAV library. All raw sequence data were deposited in the NCBI Sequence Read Archive under the BioProject ID PRJNA1224956.

### NGS read data processing, variant calling, and genotyping

**Figure 1:**
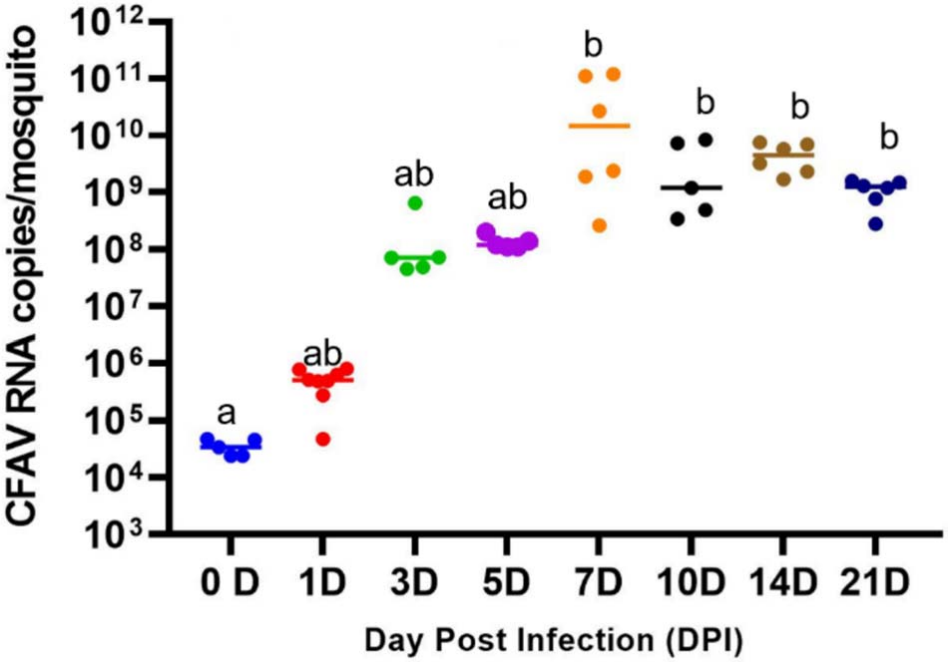
CFAV RNA levels were quantified at different time intervals in female *Ae. aegypti* mosquitoes, each dot representing a single mosquito (five dots at 0D represent five individuals), different letters above dots indicating statistically significant pairwise differences between time points, and shared letters represent no significant difference, bars showing the mean.

Reads were trimmed to remove Illumina adapter sequences using Fastp v0.23.4, maintaining an average base quality score of 25; poor quality of 5 and 50 bases in the tail of read 1 and read 2, respectively, were trimmed, and reads shorter than 150 bases were discarded. The remaining reads were aligned to the reference CFAV genome sequence (NCBI database ID: LR596014.1) using BWA-mem2 v2.2.1. The aligned file was sorted and indexed using samtools v1.19.2. Primers were trimmed from the aligned reads using iVar (Grubaugh et al. 2019). CFAV genome coverage and sequencing depth were assessed using samtools v1.19.2 (Supplementary File 1 XLSX and Supplementary Fig. 2). Single nucleotide variants (SNVs) were called using iVar with a frequency threshold of 0.01 (Grubaugh et al. 2019, Roder et al. 2023) based on an estimated CFAV RNA copy number of ~35 000 for initial cDNA synthesis (Fig. 1) according to the protocol of Grubaugh et al. 2019. The breadth of the CFAV genome of each sample was estimated at a depth cutoff of 400× (Supplementary File 1 XLSX) (Grubaugh et al. 2019). The variant at position 715 on the genome was consistently found in all samples, including the ancestral samples, at a frequency greater than 0.8, and was therefore considered the consensus sequence subsequently used in the CFAV reference genome.

We applied a stringent SNV frequency threshold for downstream population genomics analysis, filtering out SNVs that were potentially false positives or under possible artefact effect based on the following criteria: (i) false positive SNVs (*P* value > .05), (ii) <400× nucleotide base depth, and (iii) SNVs in the protein coding region that did not have codon information (insertion). We also filtered out SNVs presented in less than two samples. By requiring an SNV to appear in at least two samples, we greatly reduce the chance of retaining artefacts that slipped past filtering in a single sample. While it is true that some real SNVs might be unique, the trade-off often leans toward higher specificity—prioritizing confidence in reproducibility across the dataset rather than maximum sensitivity to every individual change. The frequency of SNVs was determined based on the deviation from the frequency of ancestral samples (the mean frequency of technical replicates) (Grubaugh et al. 2019). For example, if the frequency of SNVs was found to be 3% in the ancestral population and 5% in the post-infected mosquito population, the frequency of SNVs was corrected to 2% (i.e. 5%–3%). SNVs with a lower frequency in the mosquito population than that in the ancestral population were excluded. The frequencies of post-infection SNVs that arose after infection i.e. differed from the position of ancestral SNV sites were used as they were, without correction and defined as unique SNV sites.

### Genetic diversity analysis

We first estimated genetic complexity (the uncertainty associated with randomly sampling an allele) using the Shannon entropy (${S}_n$) formula (Grubaugh et al. 2019):


$$ {S}_{n=}\ \frac{-\left( pln(p)\right)+\left(\left(1-p\right)\times \ln \left(1-p\right)\right)}{\ln (2)} $$

where, $p$ is the minor allele frequency at each SNV. All SNVs were found to represent a single alternative minor allele. The mean ${S}_n$ of all SNV sites in each sample was used to determine the intra-host population complexity (Supplementary File 1 XLSX). Heterozygosity was calculated according to following formula (Hartle and Clark 1997):


$$ H=2\times p\times \left(1-p\right) $$

where, $p$ is the minor allele frequency at the SNV and the mean $H$ from all SNVs was calculated for each population (Supplementary File 1 XLSX). Heterozygosity is used to measure the combined effects of genetic drift and natural selection on genetic diversity.

Genetic diversity was assessed by the natural selection using ${d}_N$/${d}_S$ ratio which is the ration between the number of nonsynonymous sites per nonsynonymous site (${d}_N$) over the number of synonymous substitutions per synonymous sites (${d}_S$) of the complete CFAV coding region using the following formula (Lequime et al. 2016):


\begin{align*}{d}_S=\frac{-3\times \ln \left(1-\frac{4\times \frac{S_d}{S_s}}{3}\right)}{4}\ \textrm{and}\ {d}_N=\frac{-3\times \ln \left(1-\frac{4\times \frac{N_d}{N_s}}{3}\right)}{4} \end{align*}

where, ${S}_d$ is the number of synonymous substitutions in the sequence, ${S}_s$ is the number of the synonymous sites, ${N}_d$ is the number of nonsynonymous substitutions in the sequence, ${N}_s$ is the number of the nonsynonymous sites. ${N}_d$ and ${S}_d$ were calculated for each sample as the sum of SNV frequencies (Lequime et al. 2016). Number of the synonymous and nonsynonymous sites from the reference consensus sequence were estimated to be 2342 and 7684 respectively using SNAP tool (Korber 2000). When ${d}_N$/${d}_S$ ratio is > 1 is interpreted as the evidence of positive selection, a ${d}_N$/${d}_S$ ratio is < 1 is interpreted as negative (purifying) selection and when it is equal to 1, interpreted as neutral evolution.

### Estimation of the proportion of synonymous and nonsynonymous single nucleotide variant sites

We estimated the number of synonymous substitutions per synonymous sites (${d}_S$) and the number of nonsynonymous substitutions per nonsynonymous sites (${d}_N$) for the structural and NS genes in each population. The bioinformatics tool iVar can report not only the site frequency of SNVs but also classify them by mutation type (synonymous and nonsynonymous changes), with associated codon and amino acid information through reference-based annotation. Nonsynonymous SNVs were defined as alleles with amino acid changes from the reference genome, and synonymous SNVs were defined as alleles with no amino acid changes. The proportions of synonymous and nonsynonymous SNVs in structural genes (i.e. consisting of C, prM, M, and E genes) and NS gene (i.e. consisting of NS1, NS2A, NS2B, NS3, NS4A, NS4B, and NS5 genes) were estimated, respectively, for each replicate per sample by counting the total number of observed unique synonymous and nonsynonymous SNVs per gene (i.e. structural or NS) synonymous or nonsynonymous SNV sites. We assumed each SNV site represents a CFAV mutant variant. The synonymous and nonsynonymous sites per gene were estimated using the SNAP tool (http://www.hiv.lanl.gov; Korber 2000). The number of synonymous and nonsynonymous sites was estimated to be 512.67 and 1602.33 for structural gene and 1829.33 and 6081.67 for NS gene, respectively. The proportions of unique synonymous and nonsynonymous SNVs per gene (i.e. C, prM, M, E, NS1, NS2A, NS2B, NS3, NS4A, NS4B, and NS5 genes) were extracted and calculated from the data of whole viral populations by counting the number of synonymous and nonsynonymous SNV sites. The calculation was normalized by per Kb nucleotide.

### Statistical analysis

All the statistical analyses were performed using RStudio (v.4.4.1) with various packages including ggpubr (v.0.6.0) (Kassambara 2023). Figures were generated using the package ggplot2 (v.3.5.1) (Wickham 2016). A non-parametric Kruskal–Wallis multiple comparison test was performed to determine significant differences in CFAV population sizes over time after acute infection using GraphPad Prism version 9.5.1. One-way analysis of variance (ANOVA) with Tukey’s honestly significant difference *post hoc* test was performed to compare genetic diversity between populations using the R package multcompView (v. 0.1.10) (Graves et al. 2024). To examine the relationship between genetic diversity and the population size of CFAV or ${d}_N$/${d}_S,$a linear mixed-effects model test was conducted in RStudio using the lme4 (v.1.1.36), lmerTest (v.3.1.3) and boorm.mixed (v. 0.2.9.6) packages. Significant differences in the number of unique SNV sites per gene were tested using Fisher’s exact test, and *P*-values were adjusted using the Benjamini–Hochberg method. These analyses were performed using the built-in stats package (v.4.4.1) in RStudio. The distribution of SNV sites in the CFAV genome and plots of allele frequencies were visualized using the Python (v.3.9) package matplotlib (v.3.9.2).

To investigate changes in the genetic structure of the CFAV populations over time following acute infection, we calculated the Sorensen distance based on the presence or absence of each SNV allele across populations and performed non-metric dimensional scaling (NMDS) on the distance matrix. This analysis was performed separately for synonymous and nonsynonymous SNV sites, respectively. The Sorensen distance calculation, NMDS, and PERMANOVA test for significant differences between populations were performed using the RStudio (v.4.4.1) package vegan (v.2.6.6.1) (Oksanen et al. 2024). The data of the two allele matrices of the genetic composition are shown in the supplementary material (Supplementary File 1 XLSX).

## Results

### Population sizes of cell-fusing agent virus after an acute infection

Following an initial intrathoracic injection of CFAV (initial load: 3.5 × 10^4^ copies/mosquito), CFAV RNA copies showed a dramatic increase from 0 to 5 dpi. By 7 dpi, the viral load had reached a statistically significant (*P* = .0004) peak of 3 × 10^10^ CFAV RNA copies/mosquito—~86  000 times higher than the initial viral load. The elevated viral state persisted consistently at 10, 14, and 21 dpi (the end of the experimental period; Fig. 1) suggesting that CFAV entered a plateau phase of viral growth after 7 dpi. During this plateau phase, the average CFAV RNA copy number was 9.8 × 10^9^. These results indicated that CFAV replicated rapidly and maintained a persistently high viral load in *Ae. aegypti* mosquitoes after acute infection.

### Single nucleotide variant sites along the whole genome of cell-fusing agent virus

**Figure 2:**
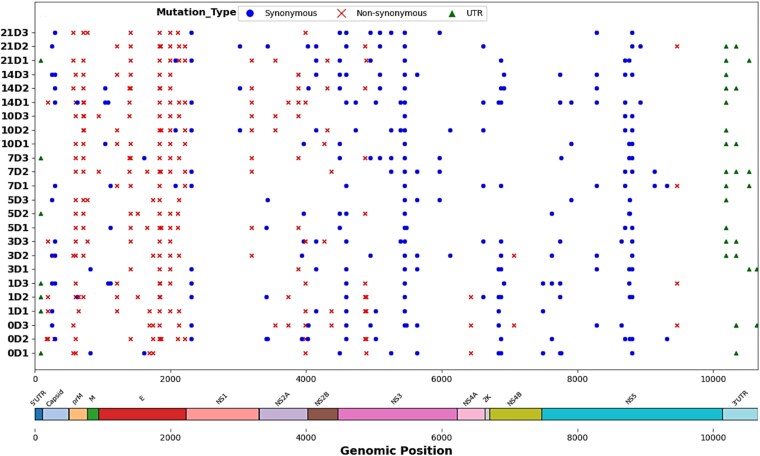
Distribution of the total 95 SNV sites identified from all samples, where the left side lists biological triplicate samples (e.g., 0D1, 0D2, and 0D3 from the 0D sample) ranging from 0D to 21D, the lower bar represents the complete CFAV genome with each gene labeled, including the 5′-UTR and 3′-UTR, and distinct symbols (circles, crosses, and triangles) indicate synonymous, nonsynonymous, and UTR SNV sites, respectively. Each symbol corresponds to the corresponding position on the CFAV genome bar. The total number of the symbols along the name of each sample represent the total number of SNV sites.

A total of 95 SNV sites were identified in all viral populations, distributed unevenly along the CFAV genome (Fig. 2). Of these, 49 were synonymous, and 40 were nonsynonymous, located in the coding regions. In addition, one SNV was detected in the 5′-UTR, and five were identified in the 3′-UTR. The average number of SNV sites identified in the CFAV genome from 0 to 21 dpi was 21.2. No SNV sites reached the consensus frequency threshold (>0.5) (Supplementary Fig. 2). A total of 68 SNV sites were identified as unique SNV sites.

### **Correlations between genetic diversity and population size of cell-fusing agent virus and**  ${\boldsymbol{d}}_{\boldsymbol{N}}$**/**${\boldsymbol{d}}_{\boldsymbol{S}}$

**Figure 3:**
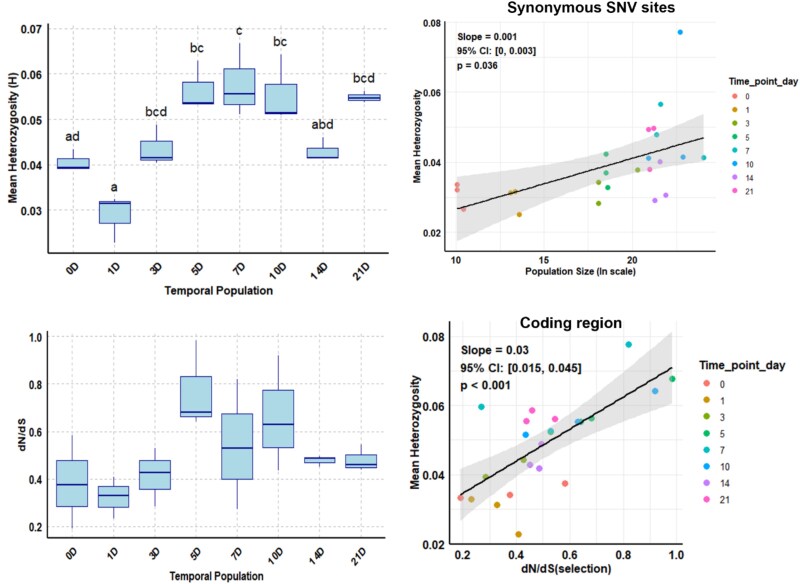
Mean heterozygosity (*H*) across all SNV sites of CFAV populations from 0 to 21 dpi (top left) and the correlation between mean heterozygosity (*H*) (across synonymous SNV sites) and CFAV population size (top right). The ${d}_N$/${d}_S$ ratio of coding region of CFAV population from 0 to 21 days post injection (bottom left) and the correlation between mean heterozygosity (coding region) and ${d}_N$/${d}_S$ (bottom right) are also shown. Different letters above each box mean statistically significant pairwise differences (*P* < .05) and shared letters denote insignificant differences, and same-coloured dots represent the population size (top right) and *d_N/d_S* (bottom right) respectively for the biological triplicate samples.

The mean value of heterozygosity (*H*) for all 95 SNV sites increased significantly with the number of days passed since inoculation. In particular, a significant increase in heterozygosity was observed between 0 and 5 dpi, 0 and 7 dpi, and 0 and 10 dpi (*P* = .02, .01, and .04, respectively) (Fig. 3, top left panel). Similarly, heterozygosity was significantly higher at later time points (5, 7, 10, and 21 dpi) than at 1 dpi (*P* = .0001, .0001, .0002, and .0003, respectively) (Fig. 3, top left panel). Shannon entropy (${S}_n$) showed a significant increase only between 1 and 5 dpi (*P* = .018), and between 1 and 7 dpi (*P* = .009) (Supplementary Fig. 4, left panel). These results suggest that the heterozygosity index is more sensitive than the Shannon entropy (${S}_n$) in the case of single alternative SNV sites.

There was no statistically significant ${d}_N$/${d}_S$ observed among all the population (Fig. 3, left bottom panel). The mean ${d}_N$/${d}_S$value from the biological replicate varied from 0.32 to 0.77. The overall mean across all the population was 0.50.

A positive correlation was observed between heterozygosity (*H*) and population size (*P* = .05) (Supplementary Fig. 5). No significant correlation was found between Shannon entropy (${S}_n$) and population size (*P* = .18) (Supplementary Fig. 4, right panel). However, correlation was relatively pronounced for synonymous SNV sites (*P* = .04) (Fig. 3, top right panel) compared to nonsynonymous SNV sites (*P* = .05). For synonymous SNV sites, the correlation between genetic diversity and population size was stronger (*H*: *P* = .04; ${S}_n$: *P* = .03) (Fig. 3, top right panel and Supplementary Fig. 7, left panel) than for nonsynonymous SNV sites (*H*: *P* = .05; ${S}_n$: *P* = .18) (Supplementary Figs 6 and 7, right panel).

A strong positive correlation was detected between heterozygosity and the ${d}_N$/${d}_S$ (*P* < .001) (Fig. 3, right bottom panel). When examined separately, both synonymous and nonsynonymous SNV sites also showed significant positive correlations with heterozygosity (*P* = .033 and *P* = .037, respectively) (Supplementary Fig. 8, left and right). In contrast, no significant correlation was found between ${d}_N$/${d}_S$ and population size (*P* = .133) (Supplementary Fig. 9). All the correlation results are summarized in Tables 1 and 2.

**Table 1:** Summary of mixed-effects model results testing the relationship between population size and CFAV genetic diversity for all SNV categories. The table includes slope estimates, 95% confidence intervals, and *P*-values for each SNV type (all, nonsynonymous, synonymous).

Genetic diversity	All SNV sites	Nonsynonymous SNV sites	Synonymous SNV sites
Shannon entropy	Slope = 0.001, 95% CI: [0, 0.002], *P* = .15	Slope = 0.001, 95% CI: [−0.001, 0.002], *P* = .18	Slope = 0.001, 95% CI: [0, 0.002], *P* = .03^*^
Heterozygosity	Slope = 0.001, 95% CI: [0, 0.003], *P* = .05^*^	Slope = 0.003, 95% CI: [0, 0.005], *P* = .05^*^	Slope = 0.001, 95% CI: [0, 0.003], *P* = .04^*^

Asterisks (^*^) indicate statistically significant differences (*P* < 0.05).

**Table 2:** Summary of mixed-effects model results testing the relationship between heterozygosity and ${d}_N$/${d}_S$for the coding region. The table includes slope estimates, 95% confidence intervals, and *P*-values for each SNV type (coding region, nonsynonymous, synonymous).

Genetic diversity	Coding region	Nonsynonymous SNV sites	Synonymous SNV sites
Heterozygosity	Slope = 0.03, 95% CI: [0.015, 0.045], *P* < .001^*^	Slope = 0.033, 95% CI: [0.002, 0.063], *P* = .037^*^	Slope = 0.024, 95% CI: [0.002, 0.046], *P* = .033^*^

Asterisks (^*^) indicate statistically significant differences (*P* < 0.05).

### Proportions of nonsynonymous and synonymous single nucleotide variant sites in structural and non-structural genes

**Figure 4:**
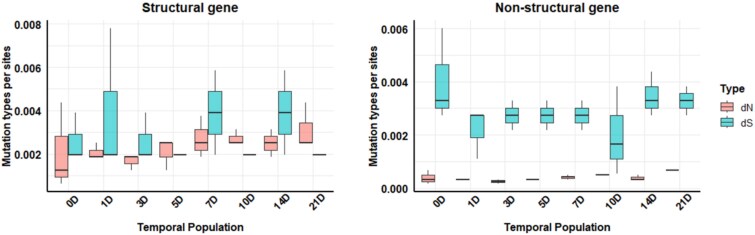
Proportion of synonymous and nonsynonymous unique SNV sites in structural and NS genes in the populations.

For the structural genes, proportion of nonsynonymous SNV sites (${d}_N$) (with amino acid changes) varied from 0.0016 to 0.003 from 0 to 21 dpi in all examined populations (Fig. 4). The proportion of synonymous SNV sites (${d}_S$) (with no amino acid changes) ranged from 0.0019 to 0.0039. The mean proportion of nonsynonymous SNV sites (${d}_N$) across all virus populations was 0.0024, which was 1.2 times lower than that of synonymous SNV sites.

Conversely, NS genes showed a predominance of synonymous SNVs (${d}_S$) over nonsynonymous SNV sites in all populations, indicating that amino acid changes were less frequent in these genes (Fig. 4). The range of synonymous unique SNV sites (${d}_S$) was from 0.002 to 0.004, whereas the range for nonsynonymous SNV sites (${d}_N$) was from 0.0003 to 0.0007. The mean proportion of synonymous SNV sites across all populations was 0.003, which was 7.14 times higher than that of nonsynonymous SNV sites.

Overall, the proportion of nonsynonymous SNV sites (${d}_N$) in structural genes was 5.8-fold higher compared to NS genes. In contrast, the proportion of synonymous SNV sites (${d}_S$) in NS genes was 1.02-fold higher than in structural genes. These results suggest there were contrasting mutation types in both structural and NS genes across all populations.

### Envelop gene contains a higher abundance of nonsynonymous amino acid changes

**Figure 5:**
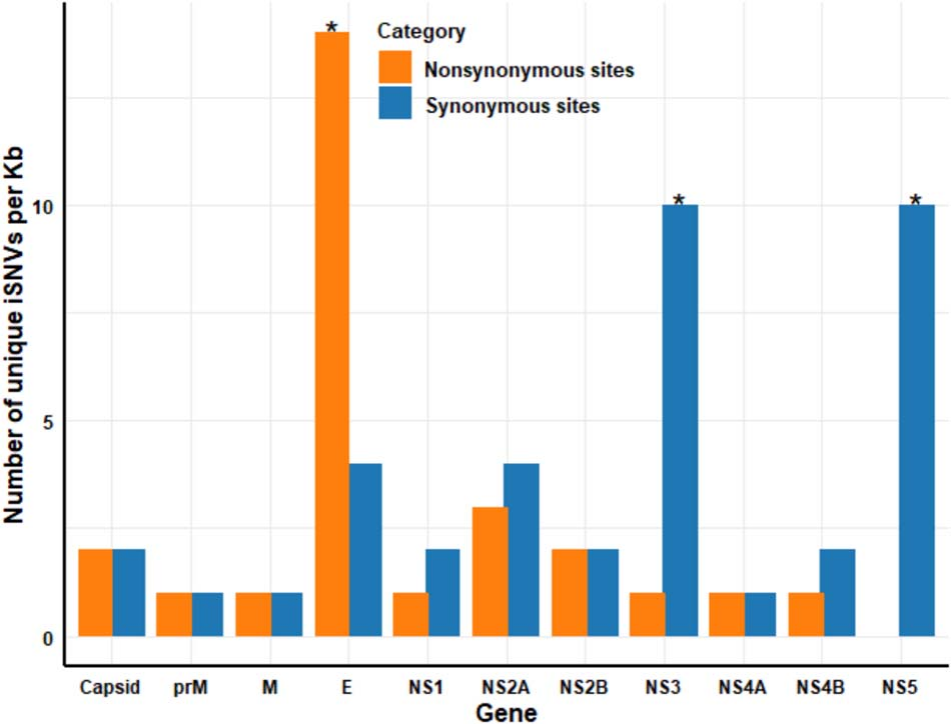
The number of unique SNV sites per gene for synonymous and nonsynonymous SNV sites, asterisk (^*^) indicates the statistically significant (*P* < .05) difference between synonymous and nonsynonymous SNV sites per gene.

Consistent with our hypothesis (3), we observed a statistically significant dominance of nonsynonymous SNV sites over synonymous SNV sites only within the E gene (prevalence: ~ 78%, *P* = 4.5 × 10^−5^), whereas similar trends were not observed in other structural proteins, including C (prevalence: 50%, *P* = 1), prM (prevalence: 50%, *P* = 1), and M gene (prevalence: 50%, *P* = 1). This result suggests that genes targeting the host cell evolve faster and are under stronger evolutionary selection (Fig. 5).

In contrast, a significant increase in the frequency of synonymous SNV sites was observed in two genes not associated with M proteins: NS3 (prevalence: ~ 91%, *P* = 4.5 × 10^−5^) and NS5 (prevalence: 100%, *P* = 1.6 × 10^−7^). However, other genes showed a lower frequency of synonymous SNV sites, such as NS1 (prevalence: ~ 67%, *P* = .64), NS2A (prevalence: 57%, *P* = .64), NS2B (prevalence: 50%, *P* = 1), NS4A (prevalence: 50%, *P* = 1), and NS4B (prevalence: ~ 67%, *P* = 1). This variation in the ratio of synonymous and nonsynonymous SNV sites between genes suggests that selective pressure acts differently on surface and M-associated proteins compared to non-M associated proteins.

### Temporal changes in the population genetic structure of the cell-fusing agent virus

**Figure 6:**
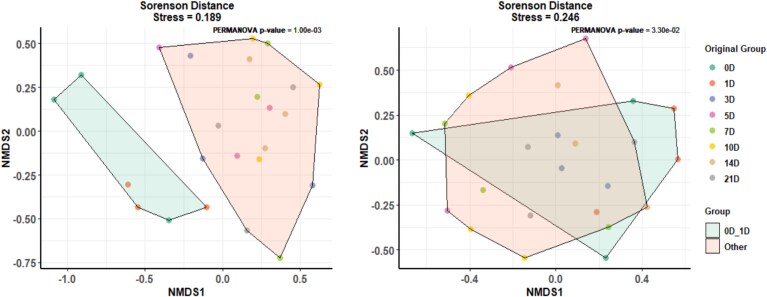
NMDS ordination plots based on Sorensen distance matrices among CFAV populations for nonsynonymous SNV sites (left panel) and synonymous SNV sites (right panel), stress value for each ordination is shown at the top centre.

Using nonsynonymous SNV sites, NMDS plot revealed two distinct clusters of genetic composition: one group consisted of viral populations from 0 and 1 dpi, while the other group consisted of populations from 3 to 21 dpi (stress: 0.189; PERMANOVA *P*-value = 1.0 × 10^−3^) (Fig. 6), indicating a shift in the genetic structure of the population after 1 dpi. However, an overlapping cluster between these two groups was observed when using synonymous SNVs, suggesting a lesser effect on the genetic differentiation between the groups. Although a marginal level of statistical significance difference was observed (PERMANOVA *P*-value = 3.3 × 10^−2^), the stress value was relatively higher (0.246) than the standard representative data.

## Discussion

### Effect of viral population size on genetic diversity under neutral evolution

In this study, we aimed to elucidate intra-host evolutionary processes and analyse the genetic diversity of CFAV, using it as an experimental model for ISVs during replication kinetics in acutely infected *Ae. aegypti* mosquitoes. Our results support the hypothesis (1) that the intra-host genetic diversity (heterozygosity/Shannon entropy) of CFAV increases with population size. Under neutral evolution, the level of genetic diversity is determined by the balance between mutation, which increases genetic diversity, and genetic drift, which reduces it (Messer 2016, Sanjuán and Domingo-Calap 2021). Genetic drift occurs typically in small population sizes. After an acute infection, the initial population size was small and is likely to experience a stronger genetic drift, resulting in a reduced overall genetic diversity. However, larger populations retained more genetic diversity due to the reduced effect of genetic drift. As the CFAV population increased rapidly at 7 dpi, it is likely that genetic drift was reduced, leading to an increase in genetic diversity.

However, genetic diversity in this study may reflect the combined effect of neutral or adaptive evolution. Genetic drift is an independent process, occurring regardless of whether evolution is neutral or adaptive. Therefore, in a small population, both synonymous and nonsynonymous SNV sites may experience a reduction in genetic diversity because of genetic drift. Synonymous mutations, which do not change the structure of a protein, are generally considered to be neutral (Gojobori et al. 1994, Lauring 2020, Li et al. 2022). Since synonymous SNV sites, which are more likely to follow neutral evolution in CFAV, the reduction of genetic diversity is likely to be the strong effect of genetic drift in the smaller population which we observed both in heterozygosity and Shannon entropy indexes (Table 1). In contrast, nonsynonymous mutations, which alter amino acid sequences, are more likely to be affected by natural selection. In a smaller population, adaptation is a rapid process in a stronger genetic drift (Cuevas et al. 2012). In the case of nonsynonymous SNV sites, the loss of genetic diversity in smaller population caused by genetic drift decreases the likelihood of favourable SNVs that confer adaptation being present in the population. Consequently, the probability of population increasing in size is also reduced. However, positive correlation between nonsynonymous heterozygosity and population size (*P* < .05) suggests that larger populations, which maintain greater genetic diversity due to reduced drift (Nakamura et al. 2018), likely to be influenced by natural selection. Note that CFAV population likely encompasses a heterogeneous mixture of mutant genomes, including singleton or multiple SNVs that may be neutral, beneficial, or detrimental. Therefore, the measurement of heterozygosity as a diversity index possibly be influenced by the genetic linkage or hitchhiking effect in the small population size.

Several researchers used ${d}_N$/${d}_S$ as a measure of the natural selection for RNA viruses and observed the dominance of purifying selection in the intra-host evolution study (Grubaugh et al. 2015, Lequime et al. 2016). Our result has also shown consistency in the dominance of purification selection across all the population—as expected in general in the intra-host diversity analysis. However, a threshold of ${d}_N$/${d}_S$ for the natural selection is relatively poorly sensitive—particularly when measured within host population (sequences are based on transient polymorphism) generally dominates purifying selection (Kryazhimskiy and Plotkin 2008, Lin et al. 2019). Because the test was originally developed for distinctly diverged genetic sequences which represent actual substitution along evolutionary lineages over a longtime scale (Kryazhimskiy and Plotkin 2008). Another benchmark for the positive selection analysis could be the presence of a site at a high-frequency level (≥ 0.5 to generate consensus sequence for a gene or complete region using the current bioinformatic tools) compared to other nonsynonymous mutation due to immune selection pressure (Parameswaran et al. 2017) which we did not observe in our frequency data. Notably, the most prominent immunity in mosquitoes is small interference RNA (siRNA) based viral inhibition (Yan et al. 2013). The generation of immune escape variant or other factors in mosquitoes is yet to be investigated.

The strong positive correlation between the heterozygosity and ${d}_N$/${d}_S$ (*P* < .001) suggests that deterministic force i.e. purifying selection played a major role in shaping genetic diversity relative to genetic drift. However, this mechanism alone is unlikely, since no significant correlation observed between ${d}_N$/${d}_S$ and population size (Supplementary Fig. 9). Instead, the significant positive association between heterozygosity with population size and ${d}_N$/${d}_S$indicates that mutation, genetic drift, and the strength of purifying selection act together to balance the overall shape of the genetic diversity. In a small population, stronger genetic drift reduces the number of both synonymous and nonsynonymous SNVs introduced by mutation, while purifying selection eliminates the deleterious variants, leading to lower genetic diversity (as observed in the 0D to 1D samples). As population size increases, the influence of genetic drift reduces, allowing overall genetic diversity to rise. In this setting, purifying selection removes only deleterious mutants from the overall mutant types while adaptive SNVs (synonymous and nonsynonymous SNVs) approaching neutral evolution contribute to an increase in overall genetic diversity.

Overall, genetic diversity in CFAV populations is likely influenced by two evolutionary processes: neutral evolution driven by genetic drift and adaptive evolution driven by natural selection. Separating the effects of these processes is challenging because genetic drift impacts all loci, including adaptive loci influenced by natural selection. Conversely, natural selection may affect neutral loci due to linkage and hitchhiking effects. In this study, both synonymous and non-synonymous SNVs showed a positive correlation with heterozygosity, population size, and ${d}_N$/${d}_S$. These results suggest that both genetic drift and natural selection contributed to genetic diversity; however, we cannot completely rule out the possibility that only one of these evolutionary processes was involved. To our knowledge, this is the first study to investigate the effect of viral population size on genetic diversity by separating synonymous and nonsynonymous SNV sites, highlighting that neutral evolution determines the effect of population size.

Furthermore, our results support the hypothesis (2) that ‘when the population size is small, the temporal change in population genetic structure is large; however, as the population size increases, this change becomes smaller.’ The population genetic structure of CFAV showed a marked difference between populations sampled on Days 0 and 1 dpi compared to those sampled from Day 3 dpi onwards. On Days 0 and 1 dpi, the virus population size was relatively small, resulting in strong genetic drift. However, after Day 1 dpi, the population size increased and remained large, leading to a weakening of the effects of genetic drift; hence, the population genetic structure is thought to have stabilized thereafter.

Although there have been no studies on the evolution of ISVs within hosts, research on viruses in mosquitoes has been conducted for several arboviruses and has yielded similar results regarding the effect of population size. For example, Lequime et al. (2016) found that the intra-host genetic diversity of DENV in the midgut of *Ae. aegypti* at 4 and 7 dpi was reduced due to rapid population decline. Grubaugh et al. (2016) explained that genetic drift and bottleneck effects significantly reduced the genetic diversity of WNV. Our findings are the first study to show that viral population size in mosquitoes affects genetic diversity and changes in population genetic structure, not only for arboviruses that can infect hosts other than mosquitoes but also for CFAVs that are specific to mosquitoes. We predicted similarities in the mechanisms of virus evolution within infected individuals, regardless of the mode of transmission to the mosquito (e.g. from mosquito to mosquito or from human or animal to mosquito), and our results confirmed this expectation.

### Synonymous and nonsynonymous single nucleotide variants in structural and non-structural genes

We found that NS genes accumulated more synonymous SNVs (${d}_S$). Since synonymous mutations do not alter protein function, they are generally considered to be evolutionarily neutral. NS proteins typically serve as components of the viral replication machinery and play crucial roles in RNA synthesis, assembly, and replication control within host cells in most *Orthoflaviviruses*, such as DENV (Kapoor et al. 1995, Tan et al. 1996, Roosendaal et al. 2006, Leung et al. 2008). Because NS proteins function intracellularly, they are not directly exposed to the host cell surface during viral entry. This results in weaker natural selection pressure, making it easier for synonymous mutations that do not change the amino acid sequence to accumulate (Domingo 2020).

In contrast, structural genes, including surface and M-associated proteins (such as C, E, prM, and M), exhibit 5.8-fold higher accumulation of nonsynonymous SNV sites (${d}_N$) compared to NS genes. This finding suggests that these structural proteins are under strong selection pressure due to their functional role and exposure to the external environment. For example, the route of DENV infection in the mosquitoes is first through the midgut *via* a bite from an infected host, followed by haemolymph and then saliva (Öhlund et al. 2019). Since the cell surface molecules of each infected tissue are different, DENV encounters tissue-specific barriers (Cruz-Oliveira et al. 2015). An example of evolutionary adaptation to this barrier is provided by Yadav et al. (2023), who found that a mucin-related protein in the midgut interacts with DENV2 virus particles, facilitating their entry into cells. Koh et al. (2021) reported that following intrathoracic injection of Palm Creek Virus, an insect-specific flavivirus, into *Ae. aegypti*, infection spread to various tissues, including midgut, ovary, and remaining tissues. It is speculated that following thoracic infection, the structural proteins of CFAV were under selective pressure to adapt to the cell molecules of different tissues within the host, leading to the accumulation of nonsynonymous mutations. This adaptive evolution may enhance binding specificity to targeted host cell surface molecules and improve tissue-targeted replication. Future research is needed to identify viral receptors that are adapted to different types of host tissue cells.

In genes involved in host cell interactions, particularly the E gene, a higher frequency of nonsynonymous SNV sites was observed, with a prevalence of 75% (*P* = 4.5 × 10–5) compared to synonymous SNV sites. Genes with frequent nonsynonymous mutations are often under positive selection, which can confer evolutionary advantages (Zhang et al. 1998, Bazykin et al. 2006). In *Orthoflaviviruses* such as DENV, the E gene encodes significant portions of the outer M proteins that are critical for viral entry into cells (Modis et al. 2004). The E protein undergoes substantial structural changes to promote binding to host cell surface receptors and facilitate M fusion, thereby determining the infected cells (Cruz-Oliveira et al. 2015; Yadav et al. 2023). Notably, a high frequency of adaptive alleles was observed for the nonsynonymous SNV located at nucleotide 1840 of the E gene. This frequency remained consistently high after 1 dpi, with an estimated mean of 0.25 at 21 dpi (Supplementary Fig. 3). The amino acid change at this position, from glutamine to lysine, suggests that adaptive changes occurred during the infection process. The presence of numerous nonsynonymous mutations in the E gene, which is critical for cell invasion, suggests that CFAV may have gained an evolutionary advantage in host-switching activity.

A high frequency of synonymous SNV sites has been observed in the NS3 and NS5 genes. Synonymous substitutions generally do not significantly affect viral function, allowing these mutations to be randomly fixed within the gene pool (Kimura 1991). NS3 functions as a protease and helicase, while NS5 functions as an RNA-dependent RNA polymerase with methyltransferase activity. Key mutations in these genes can compromise their functions (Ngo et al. 2019). In particular, the change from serine to glycine at position 5617 in NS3 does not affect replication in mammalian cells but influences host restriction in WNV (Ngo et al. 2019). The high frequency of synonymous SNV sites in NS3 and NS5 observed in our study suggests that these genes are under low selective pressure and are instead influenced by random neutral evolutionary processes, such as spontaneous mutation and genetic drift. While NS3 and NS5 are essential for viral replication and enzymatic activity, other NS genes (NS1, NS2A, NS2B, NS4A, and NS4B) are also critical for viral survival (van den Elsen et al. 2021). The exclusive observation of high frequencies of synonymous SNV sites in NS3 and NS5 is likely to result from these random neutral evolutionary processes. However, we do not exclude the possibility of other evolutionary mechanisms, such as purifying selection. It is also plausible that genes involved in conserved functional activities are likely to experience purifying selection without altering their function emphasizing the need to investigate gene-specific evolutionary processes.

### Limitations and suggestions for future studies

A major limitation of this study is that the mosquitoes were acutely infected with high concentrations of CFAV. This concentration was chosen to minimize the impact of PCR artefacts and false positive NGS reads in downstream bioinformatic analyses. However, this approach meant that we observed the evolutionary patterns of CFAV at concentrations higher than those found in the field-collected *Ae. aegypti* mosquitoes (5.42 × 10^3^ to 8.70 × 10^6^ RNA copies/mosquito) (Martin et al. 2020). Furthermore, mosquito-specific viruses are predominantly transmitted vertically from parent to offspring. We forcibly induced acute infection *via* injection to start the study with a state where the genetic diversity of the viral population at the start of evolution (i.e. 0 dpi) was as low as possible, allowing for a clearer understanding of the viral evolutionary patterns. Although, our interpretation from the overall findings aligns with our hypothesis that intra-host viral diversity increases with the increase of population size which is jointly influenced by mutation, drift, and purifying selection. Further investigation using larger or longitudinally replicated datasets is necessary to validate and refine these observations. Nonetheless, we also observed selective pressure plausibly acting on structural genes such as the E gene. It is therefore important to investigate the evolutionary trajectories of CFAV and other ISVs occurring in natural mosquito populations. Such studies are essential to complement our understanding of ISV adaptability—particularly how genetic diversity is maintained with increasing viral population size and how ISVs infect different host cell types.

Another limitation is the technical difficulty of infecting mosquitoes with a pure, single-lineage CFAV strain (i.e. zero diversity). As a result, it was not possible to determine the starting point of virus evolution at 0 dpi. Compared to the ancestral sample (the pre-infection CFAV sample) stored at −80°C, relatively high genetic variation was observed in the CFAV present in the 0 dpi mosquito samples. This difference may be attributed to the increased activity of CFAV at room temperature, resulting in genetic variation occurring between the time CFAV was reactivated from storage at −80°C and when it infected the mosquito.

In addition, although this study examined virus evolution within a single genetic strain of *Ae. aegypti*, host-related factors such as the background of different host genomes—particularly host immunity—may also influence virus evolution. For example, the antiviral activity of EVEs against CFAV replication (Suzuki et al. 2020). They have shown experimentally that CFAV-derived EVE present in the *Ae. aegypti* genome suppress CFAV infection through the piRNA production mechanism. In our study, we used a line of *Ae. aegypti* that did not have the CFAV-derived EVEs in order to avoid the effects of these EVEs. However, we encourage researchers to compare lines with and without EVEs in the future and to investigate the effect of EVEs on the intra-host evolutionary patterns of CFAV.

## Conclusion

In this study, we investigated the evolutionary patterns of the CFAV, an ISV that infects adult *Ae. aegypti*, during the first 21 dpi, focusing on the relationship between viral population dynamics and genetic diversity. The results showed that within-host genetic diversity (heterozygosity), for both synonymous and non-synonymous SNVs, was positively correlated with viral copy number (representing population size) and ${d}_N$/${d}_S$ (reflecting purifying selection). These results suggest that genetic diversity is suppressed by genetic drift when the viral population size is small and purifying selection is likely to balance the overall genetic diversity. Significant changes in genetic structure due to genetic drift were observed up to the 3rd dpi, when the population size was small; however, the genetic structure stabilized after the 3 dpi, as the population size increased. Furthermore, NS genes showed a high frequency of synonymous SNV sites suggesting experiencing weak selection pressure, whereas structural genes, especially the E gene, was likely under strong selection pressure, suggesting that adaptive evolution was occurring.

Our findings indicate that effective population size is a critical factor influencing viral evolutionary processes. In this study, CFAV evolution appears to be primarily governed by neutral processes, with mutation and genetic drift; however, purifying selection may also strongly contribute to shaping intra-host genetic diversity. Nonetheless, immune-driven selection may be limited or episodic due to observed low-frequency variant (<0.5). Importantly, larger viral populations retain higher genetic diversity, which may provide greater potential for adaptive evolution if and when selective pressures—such as host immune responses or environmental shifts—arise. In contrast, smaller viral populations, where diversity is reduced due to stronger genetic drift, reflect the understanding about the bottleneck effect during the vertical transmission or tissue tropisms. Overall, this finding supports a model of evolutionary balance, where most viral changes are neutral, but adaptive evolution can emerge sporadically when population sizes allow, and selection pressures intensify—a concept with broader relevance for understanding the evolution of insect-specific and vertically transmitted virus.

## Supplementary Material

Supplementary_figures_veaf079

Supplementary_file_S1_veaf079

## Data Availability

It is hereby stated that all data are available on the online publishing platform Dryad (DOI: 10.5061/dryad.fn2z34v5x). The raw sequencing data are also available in the NCBI Sequence Read Archive (SRA) under BioProject ID PRJNA1224956. The data will additionally be provided upon request.
