# Supplementary material for: Intra-host evolution of cell-fusing agent virus following acute infection in Aedes aegypti mosquito
Source: Virus Evol. 2025 Sep 30;11(1):veaf079. doi: 10.1093/ve/veaf079 (PMC12553305; doi:10.1093/ve/veaf079)
Supplement: Supplementary_figures_veaf079 [file supplementary_figures_veaf079.pdf]

Supplementary figures

Intra-host evolution of cell-fusing agent virus following acute infection in *Aedes aegypti* mosquito

Mohammad Mosleh Uddin[1,2,3], Yasutsugu Suzuki[1], Dan Joseph C. Logronio[1,2], Kozo Watanabe[1]

[1]Center for Marine Environmental Studies (CMES), Ehime University, Bunkyo-cho 3, Matsuyama, Ehime, 790-8577, Japan

[2]Graduate School of Science and Engineering, Ehime University, Bunkyo-cho 3, Matsuyama, Ehime, 790-857, Japan

[3]Department of Biochemistry and Molecular Biology (BMB), Faculty of Life Science, Mawlana Bhashani Science and Technology University (MBSTU), Santosh, Tangail-1902, Bangladesh

0 dpi sample

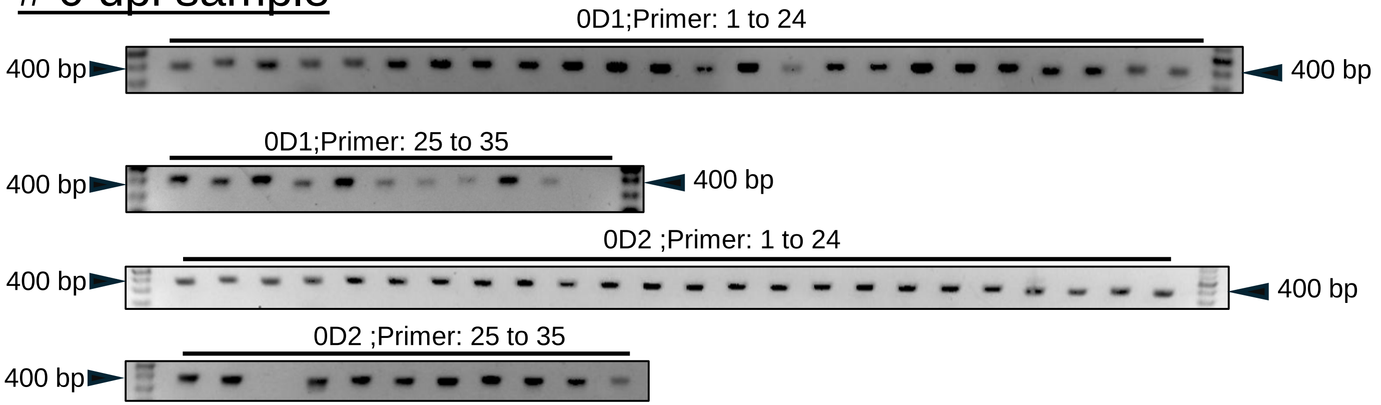

1dpi sample

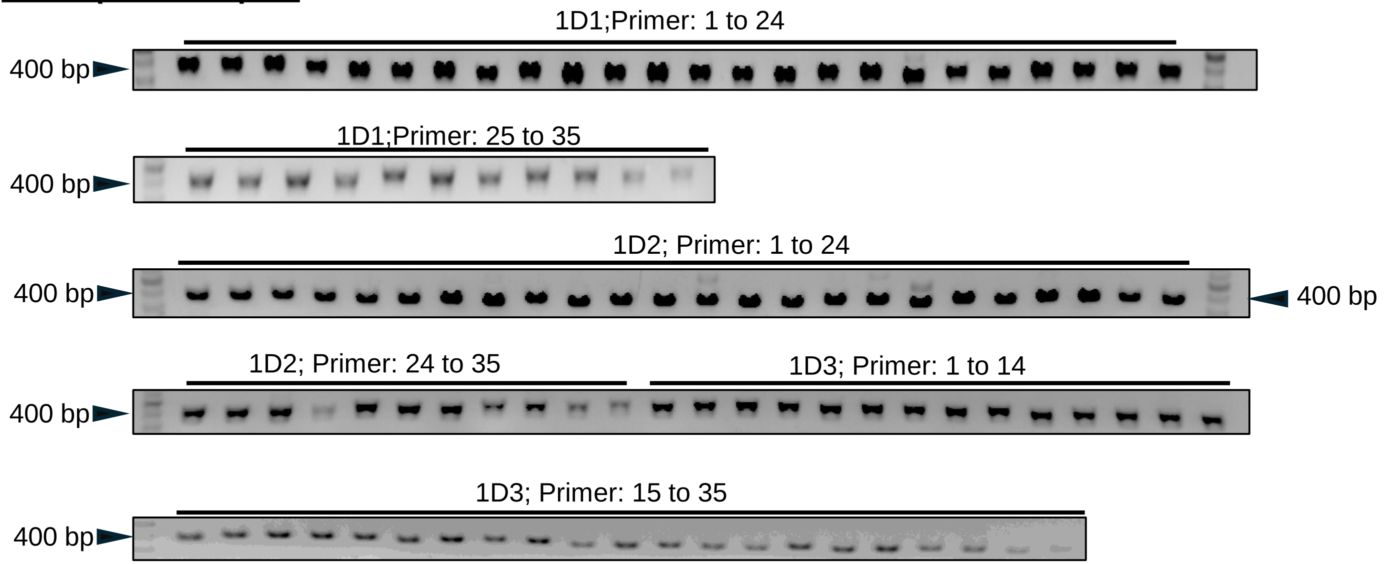

3 dpi sample

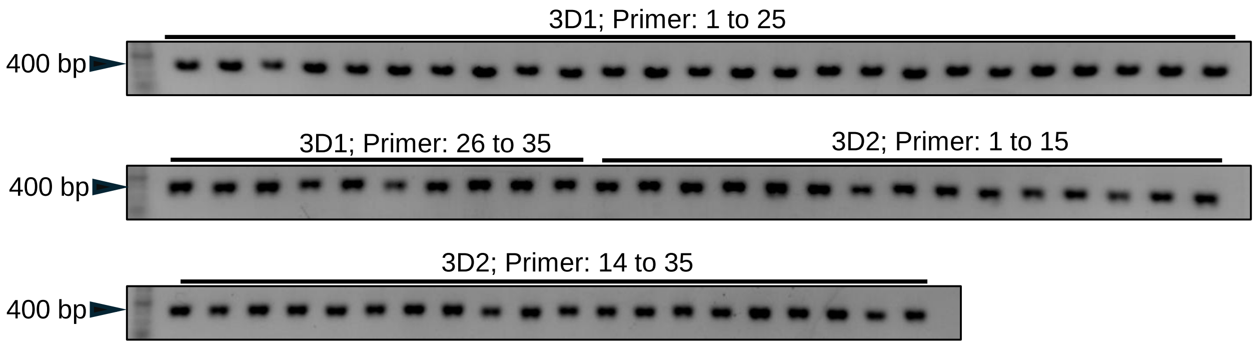

5 dpi sample

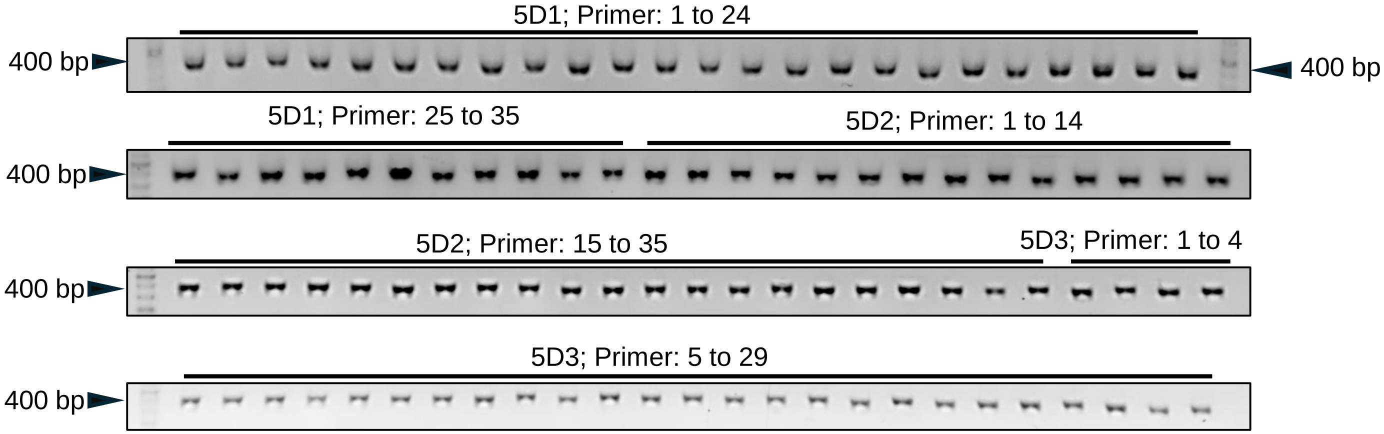

7dpi sample

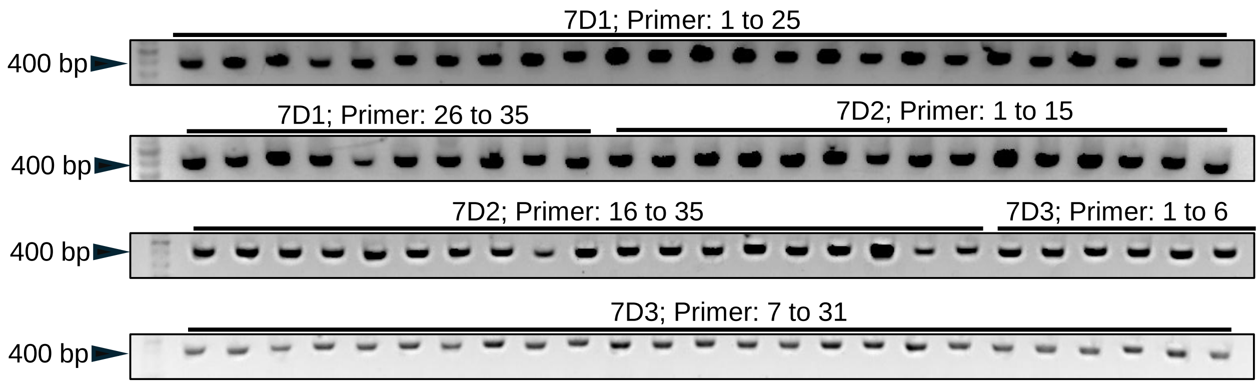

10 dpi sample

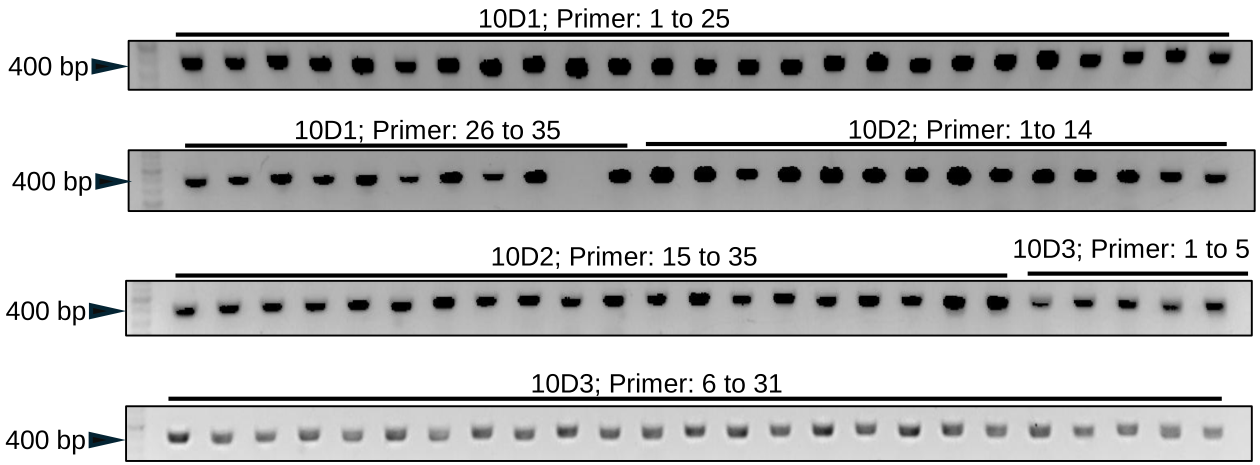

14 dpi sample

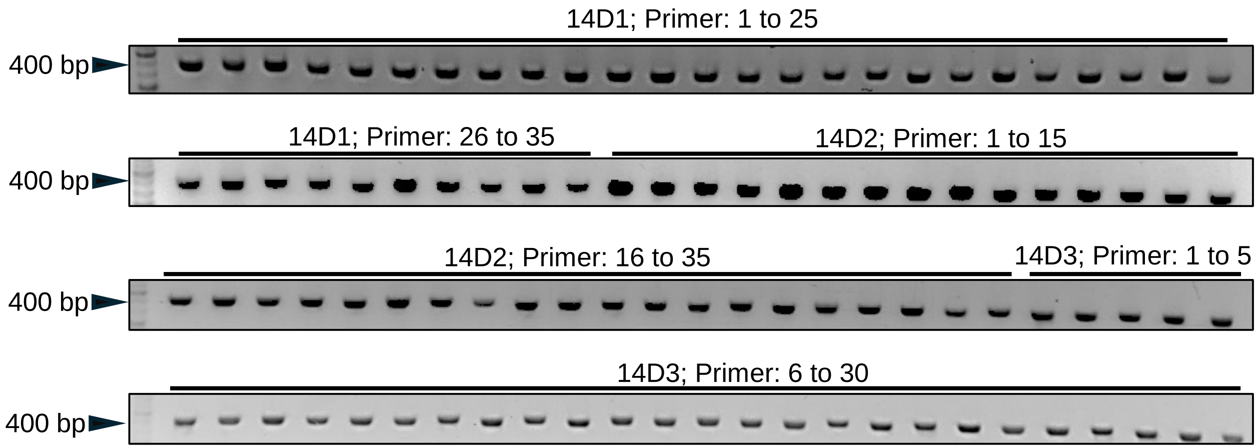

21 dpi sample

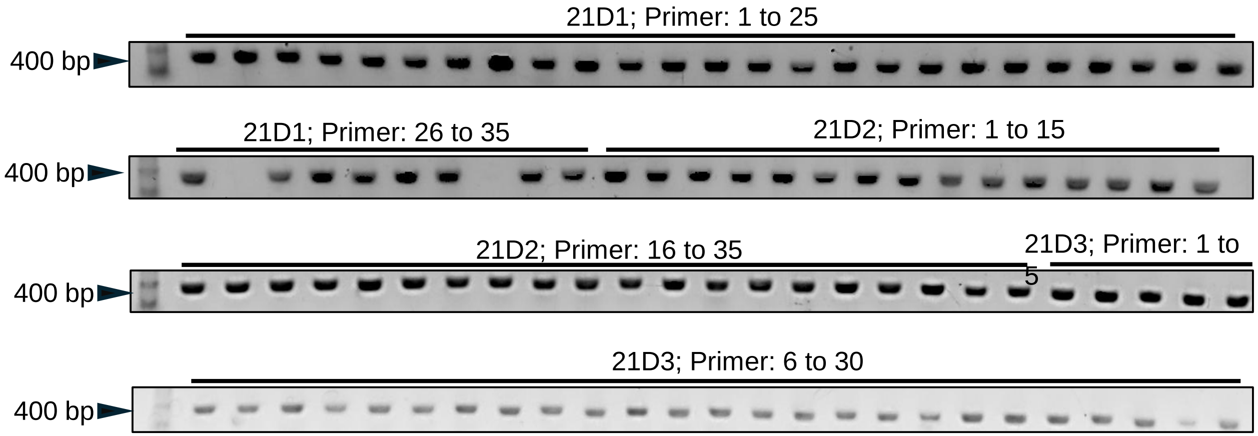

Pre-infected sample

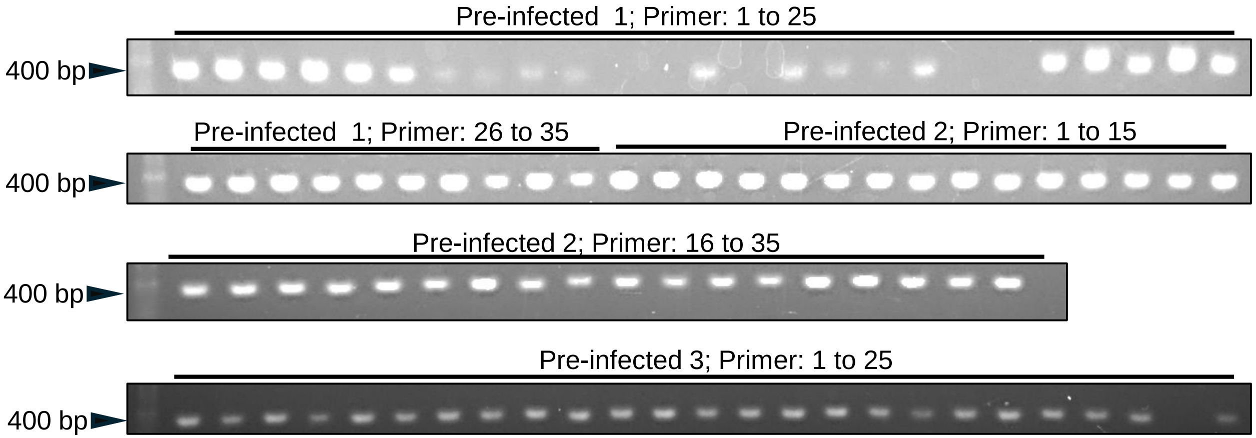

Figure S1: The representative gel pictures indicate the PCR products generated from 35 designed primers covering the entire CFAV genome. All biological triplicate samples of each time point sampling after day post infection were subjected to PCR. For instance, on the top of the gel for #1 day (1D) sample denoted as 1D1, 1D2 and 1D3 represent PCR from biological triplicates and the numerical number refers the PCR primer number from 1 to 35 which produced corresponding PCR amplicons. In some cases, PCR products from certain primers are not visible or absent in the gel. These are later confirmed by another PCR. Please also see the genome coverage and nucleotide depth of each sample (Figure S2 and File S1, XLSX). All gel pictures represent cropped images from the original.

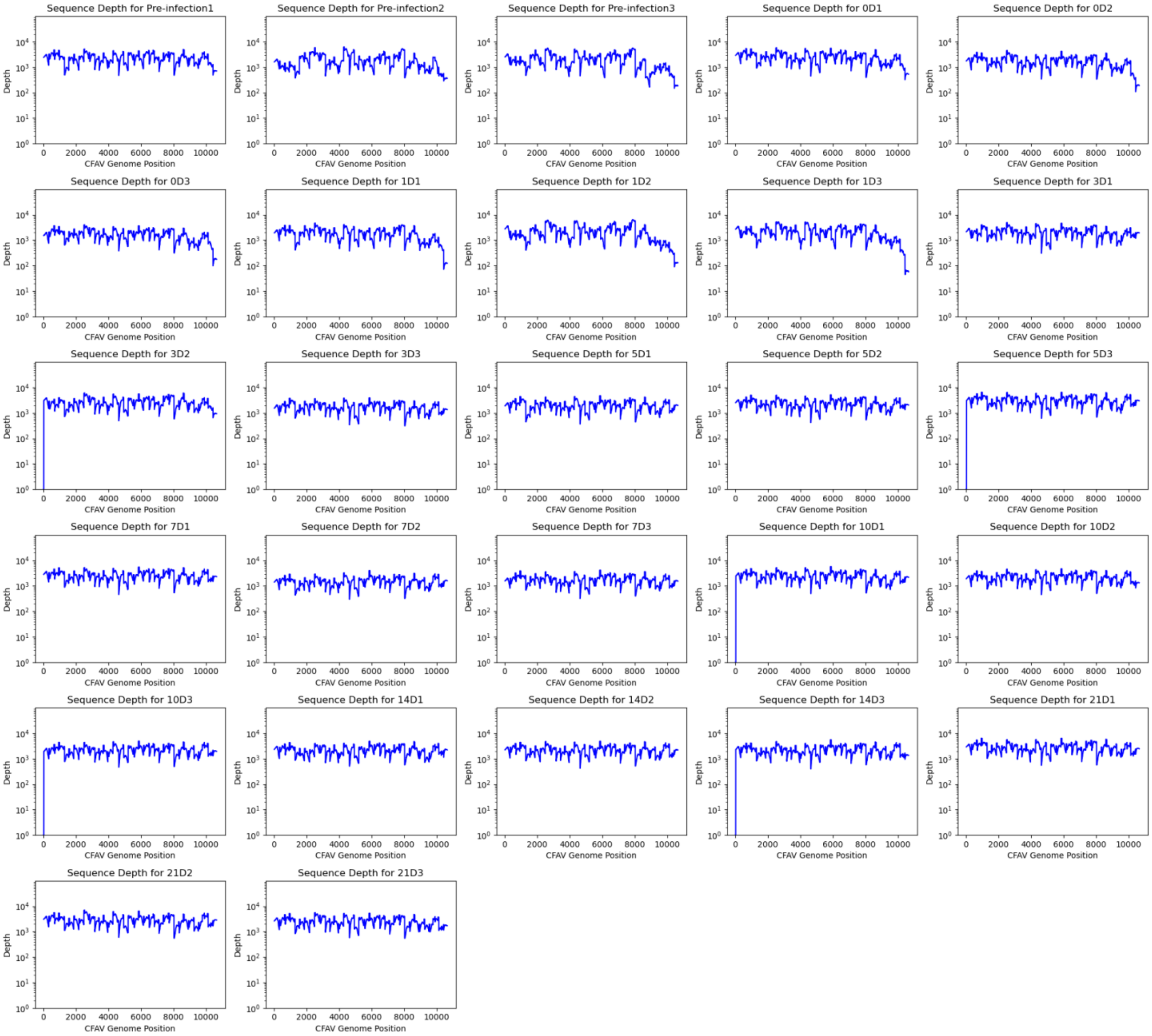

Figure S2: CFAV genome sequencing coverage and depth by sample

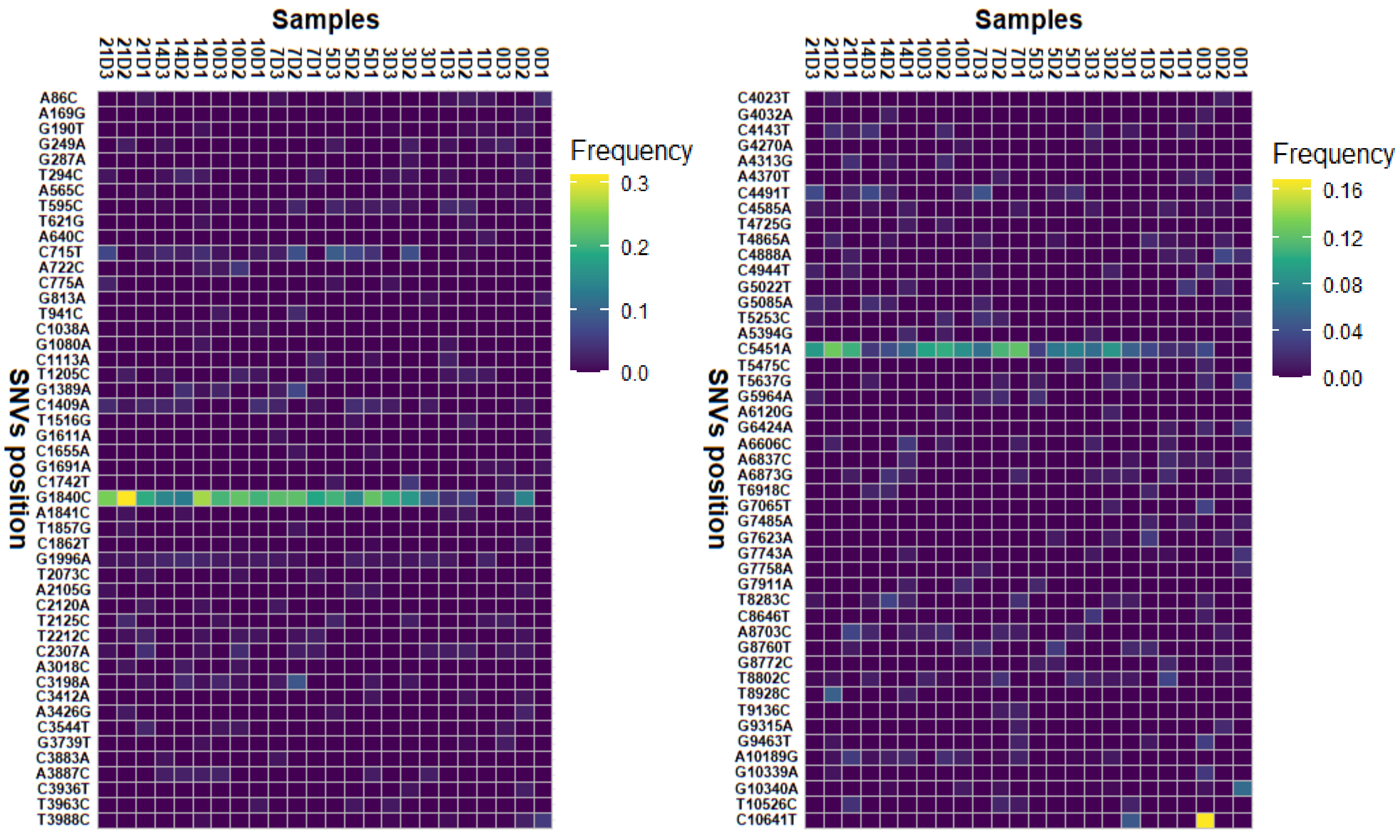

Figure S3: Characteristics of SNV sites. Heatmaps showing the alternative allele frequency (AAF) of intra-host SNV sites among samples. On the top, indicates the name of all biological triplicate samples. On the right side of both heatmaps color gradient bar represents the frequency value of AAF. The position of each SNV site from the 5’-end to the 3’-end of CFAV genome chronologically ordered in the left side of the two heatmaps. For instance, a SNV site position A86C represents ‘A’ for altered allele, ‘86’ is the genomic position and ‘C’ for reference allele.

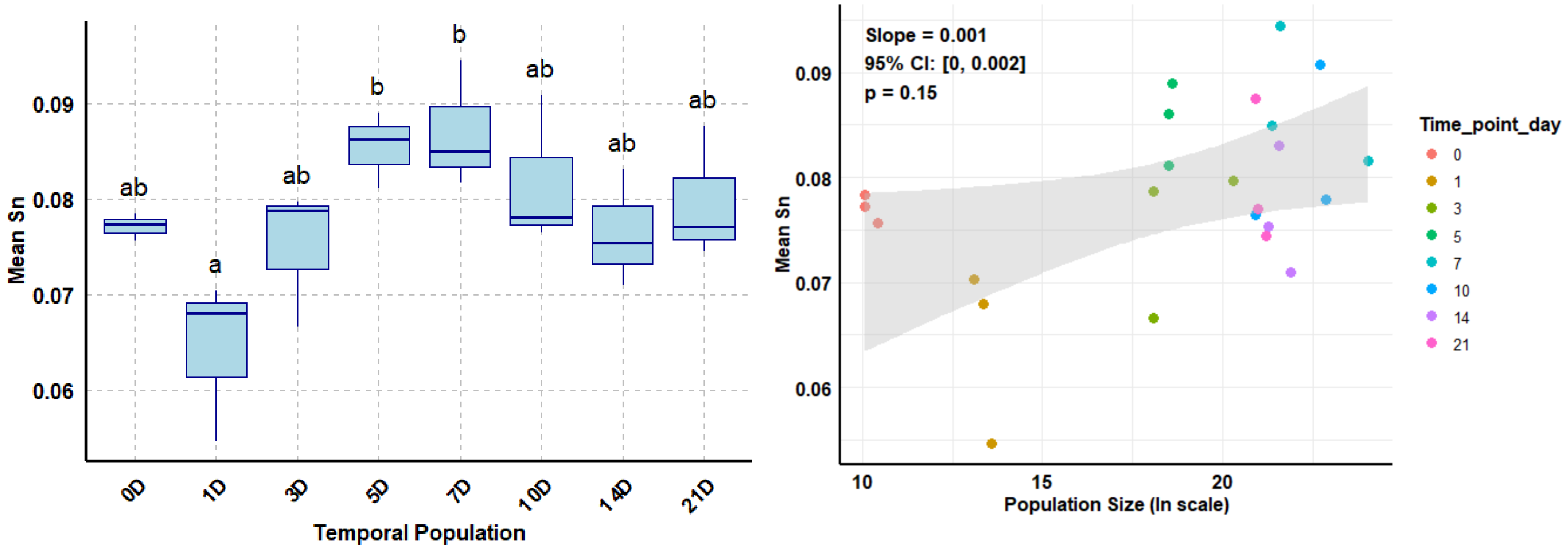

Figure S4: Mean Shannon entropy (*Sn*) across all SNV sites of CFAV populations from 0- to 21 days post injection (left) and the correlation between mean Shannon entropy (*Sn*) (across all SNV sites) and population size (right). Different letters represent the statistically significant difference ($p<0.05$). The same color dots represents the population size of biological triplicate samples.

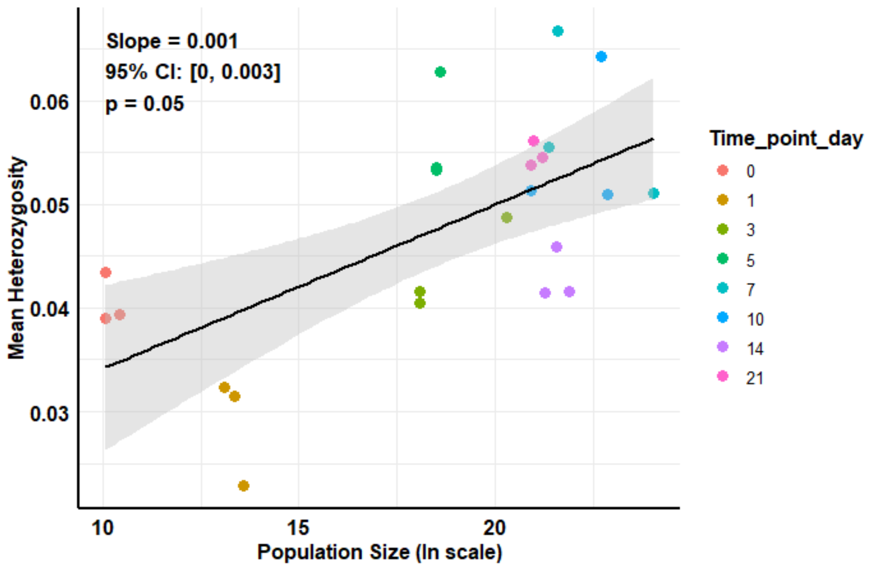

Figure S5: Correlation between mean heterozygosity *(H)* (across all SNV sites) and population size of CFAV from 0- to 21 days post injection. The same color dot points are the population size of biological triplicate samples.

Nonsynonymous SNVs

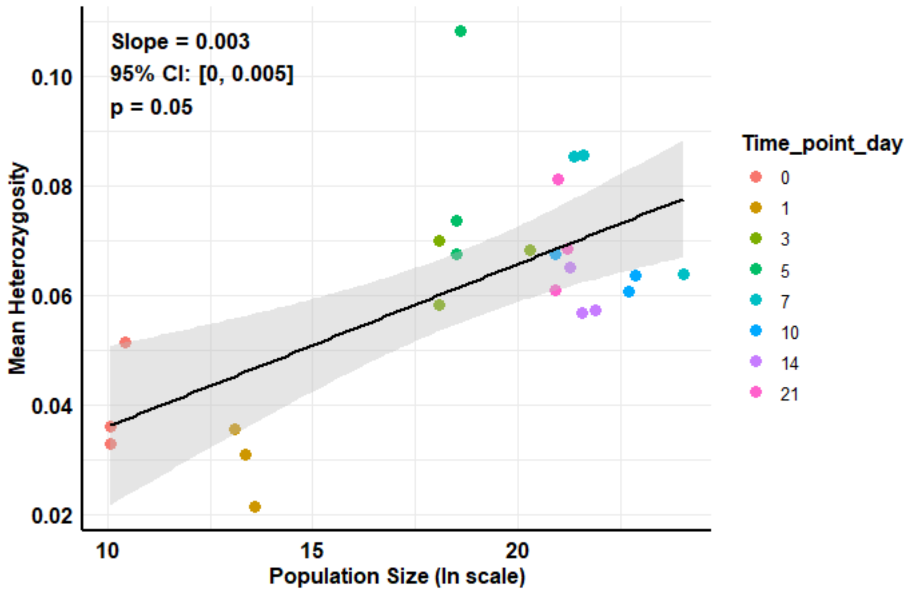

Figure S6: Correlation between mean heterozygosity (*H*) (across all nonsynonymous SNV sites) and population size of CFAV from 0- to 21 days post injection. The same color dot points indicate the population size of biological triplicate samples.

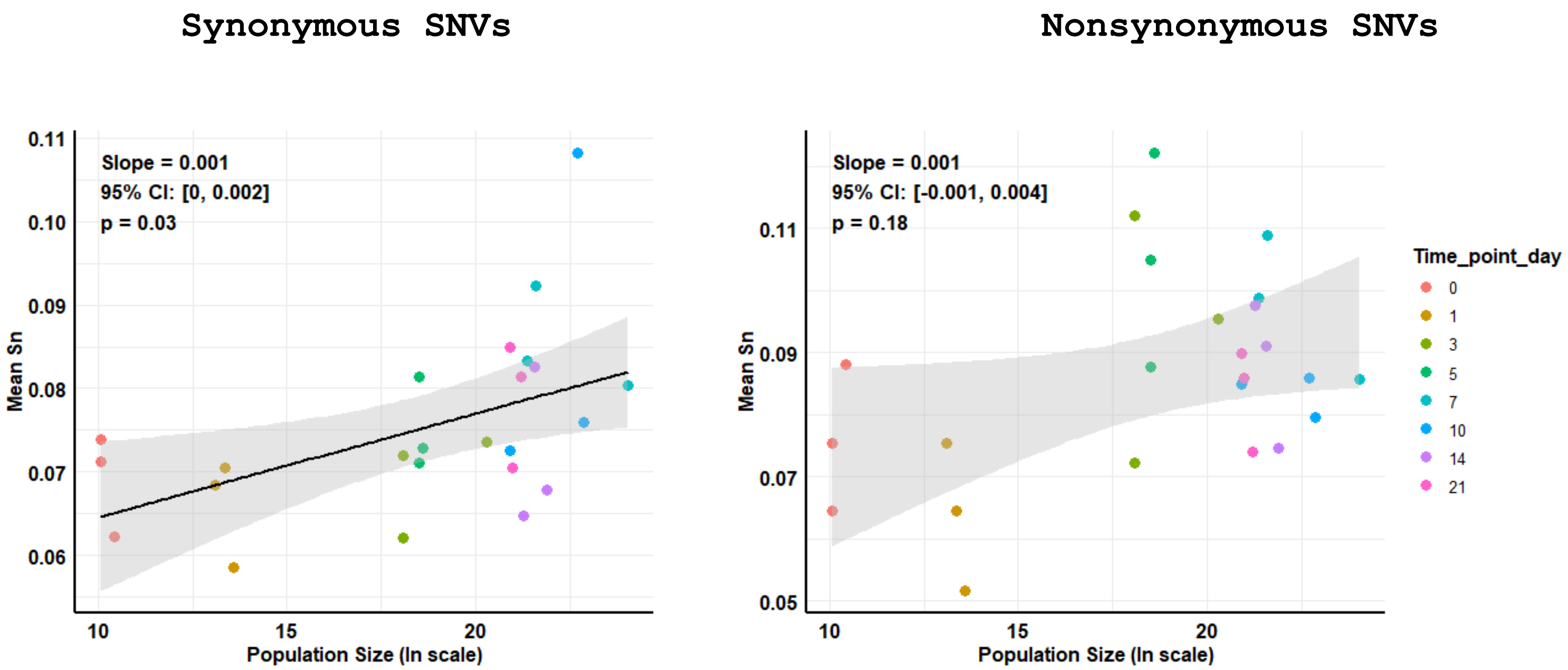

Figure S7: Correlation between mean Shannon entropy (*Sn*) (across all synonymous SNV sites (left) and nonsynonymous SNV sites (right)) with population size of CFAV from 0- to 21 days post injection. The same color dots represents the population size of biological triplicate samples.

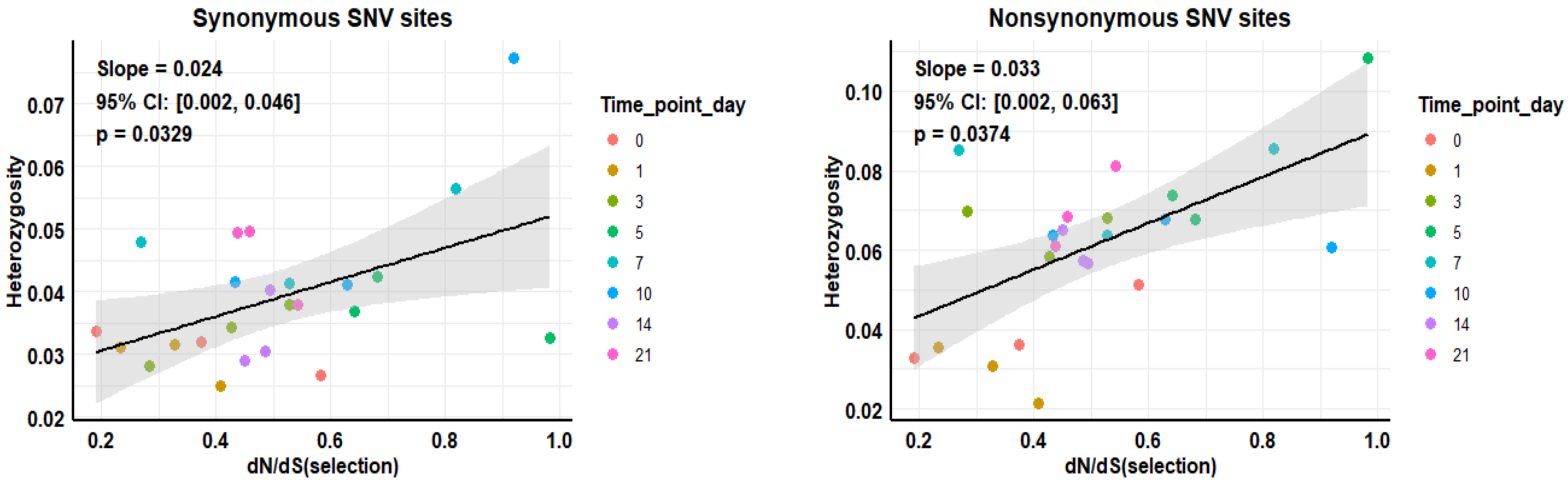

Figure S8: Correlation between mean heterozygosity (across all synonymous SNV sites (left) and nonsynonymous SNV sites (right)) and d_N/d_S of CFAV populations from 0- to 21 days post injection The same color dots represents the biological triplicate samples.

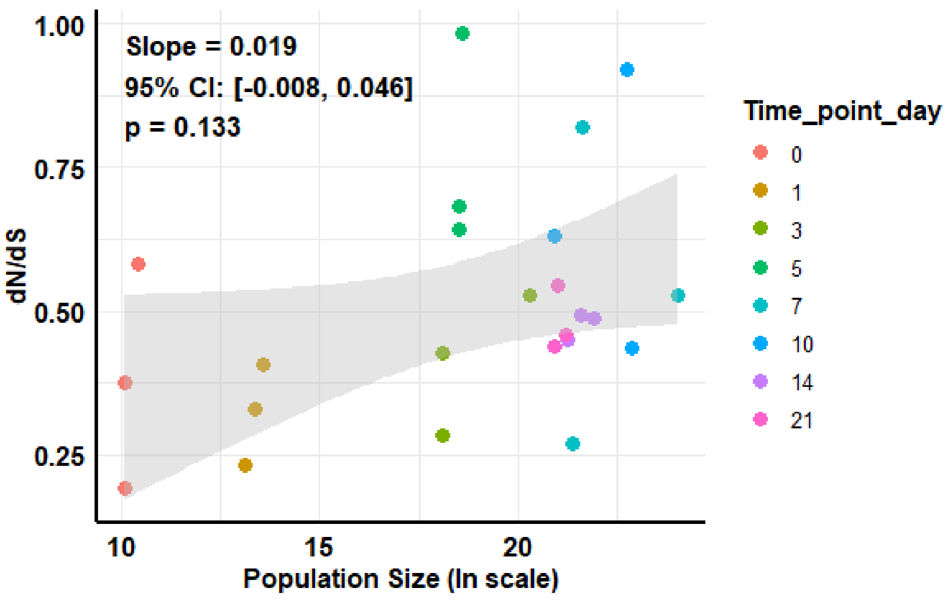

Figure S9: Correlation between d_N/d_S and CFAV population size from 0- to 21 days post injection The same color dots represents the biological triplicate samples.

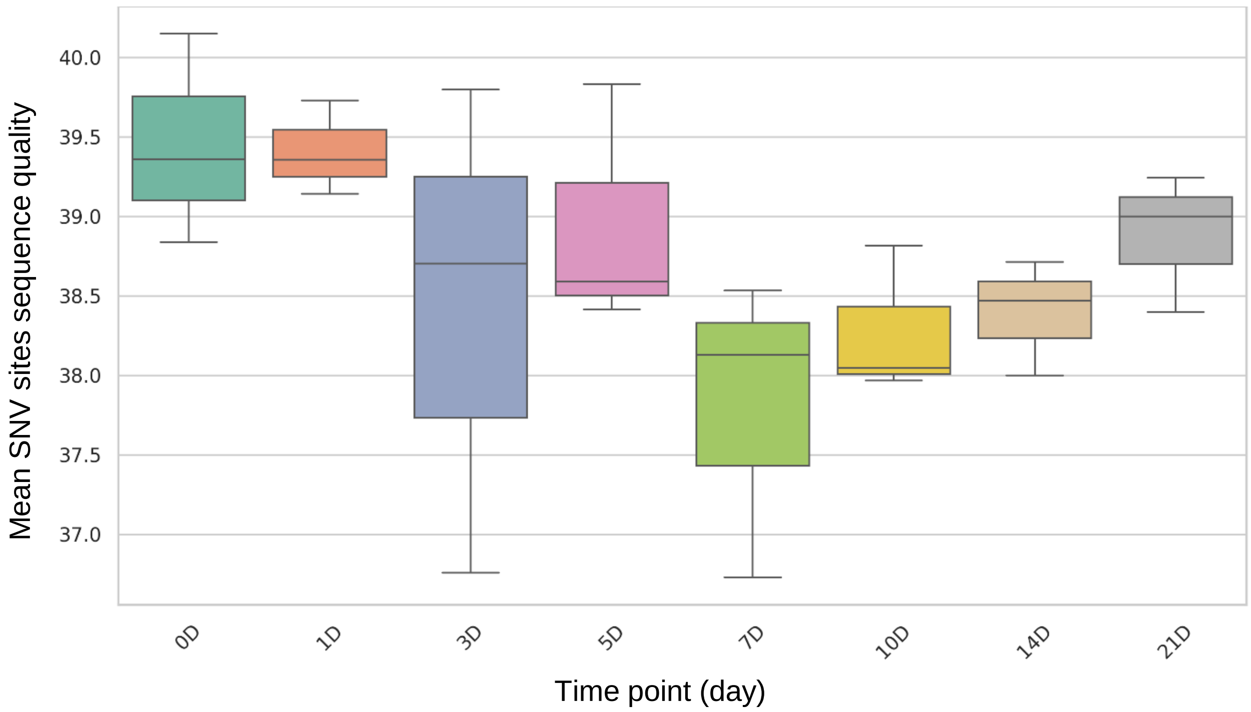

Figure S10: SNV sites sequence quality number per population. Each box bar represents the range of SNVs sites sequence quality number per biological triplicate sample. No significant difference ($p > 0.05$) across time points observed using pairwise significance test (Tukey's test).
